# Comparative Fatigue Analysis of CF-PLA Metamaterial Bone Plates for Orthopaedic Fixation

**DOI:** 10.3390/polym18101152

**Published:** 2026-05-08

**Authors:** Ani Daniel, Hamed Bakhtiari, Barun K. Das, Muhammad Aamir, Majid Tolouei-Rad

**Affiliations:** 1 Centre for Advanced Materials and Manufacturing (CAMM), School of Engineering, Edith Cowan University, Joondalup, WA 6027, Australia; h.bakhtiari@ecu.edu.au; 2 School of Engineering, Edith Cowan University, Joondalup, WA 6027, Australia; b.das@ecu.edu.au (B.K.D.); m.aamir@ecu.edu.au (M.A.)

**Keywords:** metamaterials, fatigue behaviour, 3D printing, polymers, CFRP, bone plate, FDM, CF-PLA

## Abstract

Bone plates are widely used in orthopaedic surgery to stabilise fractured bones and support healing following traumatic injuries or osteotomies. However, conventional metallic bone plates suffer from stress shielding and stiffness mismatch with bone, which can hinder optimal healing. Additive manufacturing enables the incorporation of novel metamaterial architectures into polymer-based implants to enhance mechanical properties. The fatigue behaviour of these implants during the healing period is critical to ensuring their structural integrity and long-term performance. In this study, the compressive fatigue performance of fused deposition modelling (FDM)-printed carbon fibre-reinforced polylactic acid (CF-PLA) bone plates were investigated. Four metamaterial structures—tetrachiral, re-entrant, rotating square, and hexagonal—were evaluated under strain-controlled cyclic loading at 20%, 40%, 60%, and 80% of their respective yield strains. The results showed a strong dependence of fatigue behaviour on lattice geometry. Among the tested configurations, the re-entrant structured bone plate exhibited the best overall fatigue performance, sustaining up to 100,000 cycles at moderate strain levels and showing delayed stiffness degradation under high strain conditions. In contrast, rotating square and hexagonal structures showed early stiffness loss and failure at higher strain levels. These findings highlight the importance of lattice design in fatigue performance, although FDM-induced printing defects significantly influence overall fatigue behaviour.

## 1. Introduction

Bone fractures and large skeletal defects represent a persistent and growing challenge for healthcare systems worldwide, particularly in the context of ageing populations, increasing life expectancy, and rising incidence of osteoporosis and trauma-related injuries. Global epidemiological data indicates a substantial rise in fracture cases over recent decades, resulting in significant medical, social, and economic burdens [1,2]. Although natural bone possesses an intrinsic capacity for self-healing, this ability is limited when defects exceed a critical size or when pathological conditions such as infection, metabolic disorders, or compromised vascularization interfere with the normal healing cascade [1]. Bone plates are commonly used in orthopaedic surgery to stabilise and support fractured bones following traumatic injuries or osteotomies. They provide rigid fixation, enable accurate realignment of bone fragments, and apply compressive stress and strain at the fracture site to facilitate bone healing [3,4]. Patient-specific bone plates are increasingly utilised when standard plates do not adequately match a patient’s anatomy. These customised implants provide greater precision and stability in fracture fixation, while also reducing surgical time and improving postoperative outcomes due to their superior anatomical conformity [3,5,6].

Various classes of materials have been explored for the fabrication of bone plates, including metals, ceramics, and polymers [6,7,8,9,10,11]. Traditional metallic bone plates provide excellent mechanical strength but suffer from significant limitations. Conventional stainless steel and titanium plates cause stress shielding and loosening due to modulus mismatch with bone tissue [2,12]. R. Deshmukh et al. [13] identifies additional problems, including corrosion, metal incompatibility, and decreased bone mass. Polymeric plates address these limitations through biocompatibility and tailorability [14]. Among biodegradable polymers, polylactic acid (PLA) has emerged as one of the most widely investigated materials for bone tissue engineering. PLA is biocompatible, biodegradable, FDA-approved for biomedical applications, and exhibits mechanical properties comparable to those of trabecular bone. PLA degrades primarily through hydrolytic chain scission, producing lactic acid as a degradation by-product that can be metabolised by the human body [15,16]. A variety of polymer matrices can be combined with carbon fibre (CF) through different manufacturing methods, producing composites with distinctive properties. CF implants are FDA-approved materials [17]. CF composites used in medical devices are evaluated for biocompatibility, including testing for toxicity and sensitization (ISO 10993-10-1995) [18]. In addition, CF is popular in medical devices because it is radiolucent, meaning it does not cause artefacts in CT/MRI scans and allows for radiation therapy without backscattering [19]. Wolter et al. [2] reported that CFs integrate into tissue with minimal foreign body reaction, and while they may rupture over time, connective tissue formation maintains mechanical function. CF microparticles were shown to be absorbed by macrophages or multinucleated giant cells and subsequently transported via the lymphatic system without inducing significant adverse biological responses. Carbon fibre-reinforced polymers (CFRPs) are increasingly used in medical applications as lightweight, high-strength, radiolucent implants with adjustable stiffness, offering an alternative to traditional metal implants [19,20,21,22]. There are several studies focusing on the application and advantages of polymer composite bone plates [23,24,25,26]. Studies have demonstrated that CFRPs can promote osseointegration in bone implants and, in some applications, outperform conventional titanium alloys [20,27,28]. Claes et al. [29] showed that rigid stainless-steel plates (190 GPa) caused greater bone loss through endosteal resorption than more flexible carbon fibre-reinforced carbon (CFC) plates (60 GPa). The elastic modulus of the bone beneath the CFC plate was 27% higher than that beneath the stainless-steel plate, indicating less impairment of bone strength with the more flexible plates. Similarly, Zaheer et al. [30] found that flexible CF-Nylon plates (40 GPa) promoted better fracture healing and tissue formation than stainless steel, highlighting the advantage of composite plates in supporting endochondral ossification. Composite bone plates, due to their flexible nature, supported better tissue formation by promoting endochondral ossification [30,31]. Compared with unreinforced polymers (e.g., PEEK/PLA), CF reinforcement restores the structural rigidity and fatigue resistance needed for fixation while still avoiding the very high stiffness of metals [32]. Paz-Gonzalez et al. [33] reported that the polylactic acid (PLA)/CFRP structural composite has an elastic modulus and strength that are 1.25 and 1.88 times greater than those of natural bone, respectively. Furthermore, cell viability results showed more than 80% cell proliferation in these materials.

Advancements in additive manufacturing (AM) technologies, particularly in fused deposition modelling (FDM), have enabled the fabrication of highly complex and customizable polymeric metamaterial structures [34,35]. The FDM technique operates by the use of filament as raw material. The preheated nozzle transforms the filament into a semi-liquid state. The first layer will be printed on the plate by injecting this semi-liquid according to the input from the slicing software. Subsequent layers will then be printed sequentially [36,37]. FDM remains the most popular due to its cost-effectiveness and versatility, particularly with materials like PLA and ABS, offering varying mechanical properties based on infill density and pattern.

The internal structure can significantly influence the mechanical properties of implants. The emerging concept of metamaterials presents a promising avenue for creating materials with unique or unprecedented properties and enhanced functionalities [36]. Metamaterial structures are artificially engineered structures that leverage engineered microstructures to achieve mechanical and functional properties unattainable by conventional material structures [38,39]. Metamaterials are known for their unusual mechanical properties, such as low-density, ultra-light, negative Poisson’s ratio, negative stiffness and negative thermal expansion [40,41]. They implement an alternative approach to designing the cell architectures in comparison to the traditional way of modifying the chemical compositions [42]. Auxetic metamaterials, also known as negative Poisson’s ratio materials, contract laterally when compressed and expand laterally when stretched [43]. Metamaterials designed for biomedical applications are referred to as ‘meta-biomaterials’ [4]. Its unique mechanical properties make these materials promising candidates for advanced biomedical engineering applications, including bone implants, vascular stents, and other healthcare devices [43]. Studies have shown that when the gap between fractured bones is between 2 and 4 mm, micro-movements are desirable for callus formation and facilitate the bridging of bony fragments [44,45,46]. Yamaji et al. [2] reported that 0.7 mm of micromovement accelerated bone growth when the fracture gap was small (2 mm) during the initial healing period of four weeks. The Auxetic structure has the potential to facilitate effective fixation, as its negative Poisson’s ratio enables micro-movement, resulting in fixation that provides relative stability instead of absolute stability [47]. Consequently, significant research efforts have been directed toward exploring their potential and optimising their performance for biomedical applications [48,49,50].

From a mechanical standpoint, bone and bone implants are subjected to a combination of quasi-static and cyclic loading conditions during daily activities such as standing, walking, climbing, and running. While quasi-static loads arise from body weight and posture, cyclic loads generate repetitive stresses that may lead to fatigue damage over time [51]. Fatigue failure is particularly critical because it can occur at stress levels well below the static strength of a material, making it a dominant failure mode for load-bearing implants [52]. Experimental and clinical observations have demonstrated that fatigue fractures may occur even under physiologically relevant strain amplitudes, highlighting the importance of fatigue resistance in bone plate design [51,53,54]. On average, each leg experiences one million cycles over six months at a loading frequency of 1 Hz [55]. Understanding fatigue failure mechanisms is crucial for optimising implant design and manufacturing. Many case studies show that fatigue failure is a significant concern for implants, particularly in orthopaedic and dental applications [56,57,58,59]. Fatigue failure in polymeric bone plates can occur through mechanical damage, thermal damage, or a combination of both [60,61]. Similar to natural bone, polymeric implants exhibit accumulation of residual strain and reduction in stiffness over fatigue cycles due to their viscoelastic nature [62]. Unlike metallic materials, where crack propagation dominates fatigue life, polymers typically fail due to gradual damage accumulation and crack initiation, which accounts for a large proportion of their fatigue lifespan. These mechanisms are highly sensitive to loading amplitude, frequency, temperature, and environmental conditions, making realistic fatigue assessment particularly challenging [52,63,64].

Factors such as build density, surface porosity, and strut architecture also significantly affect fatigue performance. In polymeric materials, low thermal conductivity can lead to heat accumulation during cyclic loading, causing overheating and stiffness degradation. Additionally, manufacturing defects from 3D printing, such as surface roughness, pores, voids, and cavities, as well as printing parameters like build orientation, play a critical role in crack initiation and propagation [52,55]. Previous studies have examined various factors affecting the fatigue performance of metamaterials and additively manufactured polymer structures. Francesconi et al. [65] demonstrated that auxetic metamaterial geometries exhibit more than 20% longer fatigue life than conventional positive Poisson’s ratio structures due to delayed crack initiation and reduced stress intensity during crack propagation. Ahmadi et al. [66] highlighted that, in additively manufactured meta-biomaterials, fatigue performance depends not only on the intrinsic material strength but also on the surface roughness of the struts, which significantly influences crack initiation. Additionally, Dadashi et al. [67] reported that lower printing speeds improve the fatigue life of FDM-printed PLA metamaterials by enhancing interlayer bonding, while Gomez-Gras et al. [68] found that infill density is the most influential printing parameter affecting fatigue life, followed by layer height.

The present study investigates the fatigue behaviour of FDM-fabricated CF-PLA bone plates incorporating metamaterial architectures. To the best of the authors’ knowledge, this is the first systematic study to evaluate the fatigue performance of CF-PLA with distinct metamaterial configurations for bone plate applications. The findings provide novel insights into the fatigue response of four metamaterial designs, offering valuable guidance for the optimisation of patient-specific bone fixation plates.

## 2. Materials and Methods

The CF-PLA plates examined in this study are designed as orthopaedic fixation devices, providing mechanical support during the bone healing process and progressively transferring load to the bones as the polymer matrix degrades. PLA is a widely recognised biodegradable polymer, with in vivo hydrolytic degradation occurring over periods ranging from months to several years, depending on factors such as molecular weight, crystallinity, and implant geometry, thereby making it well suited for temporary fixation applications [69,70,71]. Previous studies have shown that carbon fibre can promote osseointegration, and that resulting microparticles can be gradually transported via the lymphatic system without inducing significant adverse biological responses [28,72]. Together, these characteristics support the use of CF-PLA as a bone plate material. Our previous study [4] provides details on the design, fabrication, and mechanical characterisation of metamaterial-structured CF-PLA bone plates under static loading.

**Figure 1:**
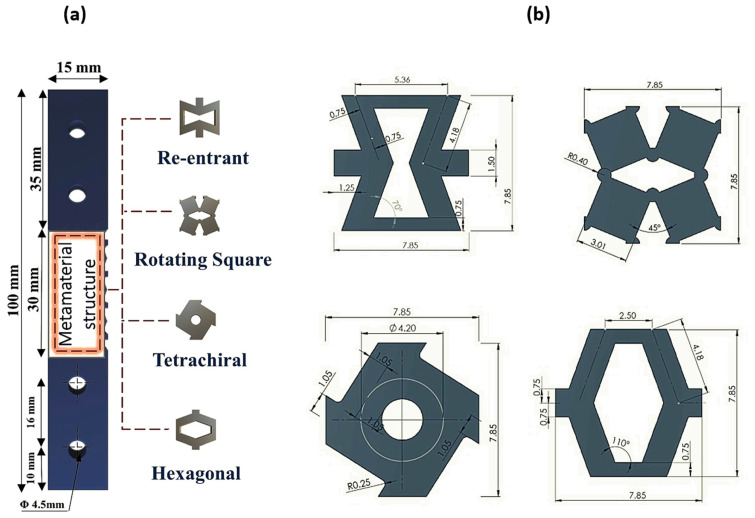
(**a**) Bone plate specimen and the selected metamaterial lattice configurations used for mechanical testing; (**b**) dimensions of the corresponding metamaterial structures. All measurements are reported in millimetres (mm).

In this study, the fatigue performance of 3D-printed CF-PLA bone plates was examined. Four distinct metamaterial architectures, namely re-entrant, rotating square, tetrachiral, and hexagonal configurations, were employed in the design of the bone plate. Figure 1a illustrates the bone plate along with the selected metamaterial configurations, while the geometric parameters of each structure are provided in Figure 1b and Table 1. Each unit cell was sized at 7.85 mm^2^, with an open area of 40%, resulting in a relative density of 60%. The re-entrant, rotating square, and tetrachiral configurations were chosen due to their extensive use in biomedical applications. These structures exhibit high anisotropy, closely resembling that of natural bone, and their geometries can be tailored to achieve a range of mechanical properties [73,74,75]. A non-auxetic hexagonal structure was also selected to compare its performance with the auxetic structures.

**Figure 2:**
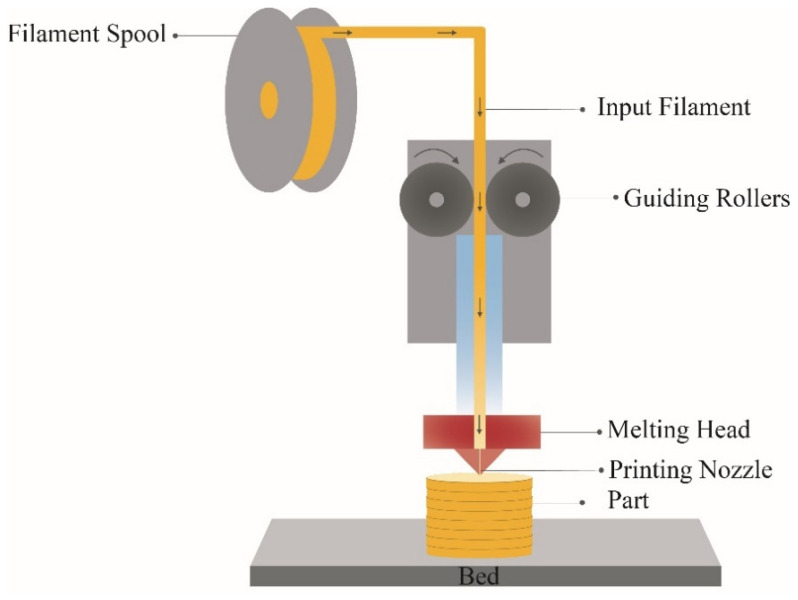
Schematic diagram of the FDM system.

**Figure 3:**
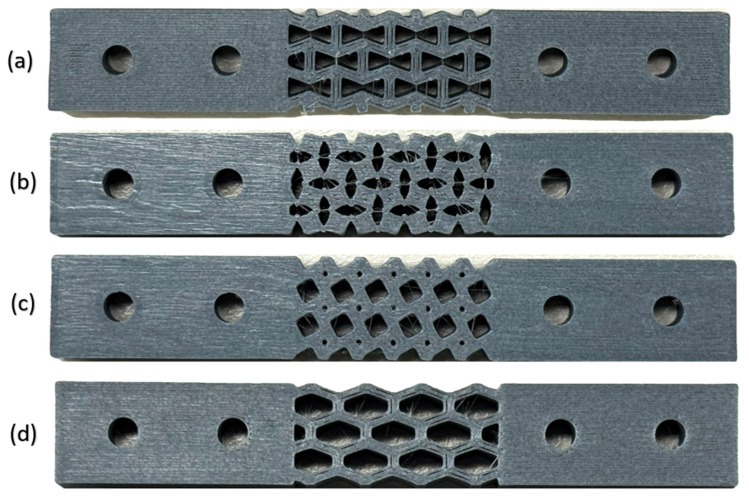
FDM printed bone plates with metamaterial structures (**a**) re-entrant, (**b**) rotating square, (**c**) tetrachiral, (**d**) hexagonal and solid ends with drilled holes to fix to the bone.

Figure 2 shows the schematic diagram of the FM process. X3D Pro CF filament (X3D, Perth, Australia) was used for this study to print bone plates. A total of 30% CF and 70% PLA by weight was the composition of the filament. FDM printer Bambu lab^®^ (Shenzhen Tuozhu Technology Co., Ltd., Shenzhen, China) was used to print bone plates. In the present study, 3D printing parameters suggested by the filament manufacturer (Table 2) were used to produce the bone plates, and all bone plates were printed in the horizontal direction. Figure 3 shows the printed bone plates with different metamaterial structures.

Fatigue experiments were performed in strain-controlled mode at strain levels of 20%, 40%, 60%, and 80% of the yield strain using an Instron 8801 testing machine. To ensure loading remained within the elastic regime, the yield strain for each specimen was determined from results obtained in prior compression tests [4], thereby preventing the applied fatigue loads from exceeding the elastic limit. Compressive results for each metamaterial bone plate are given in Table 3.

**Figure 4:**
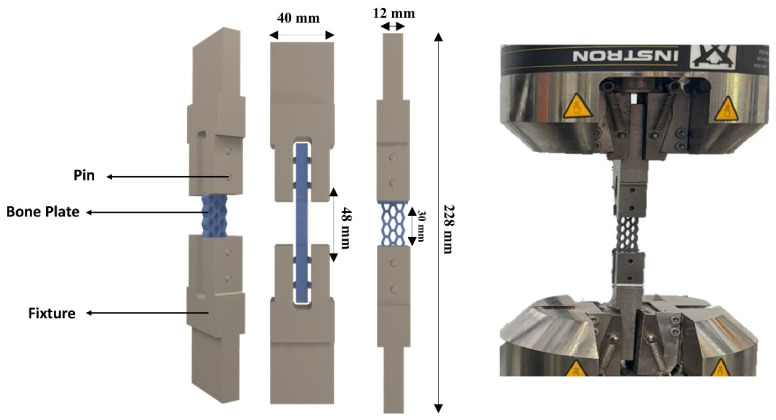
Fixture setup for fatigue testing.

For the fatigue testing, a fixture (Figure 4) was designed to replicate real bone fixation. In real conditions, these pins hold the bone plate to the fractured bone on either side.

Each bone plate was initially preloaded to approximately 0.2 MPa and subsequently subjected to 100,000 sinusoidal compressive cycles at a frequency of 5 Hz. Force and displacement data were recorded from which compressive stress, strain, and modulus were calculated for each cycle. Fatigue damage in polymeric scaffolds is typically characterised by a reduction in stiffness. According to the literature, fatigue failure in natural bone is generally associated with a stiffness reduction of 50–90% [52,55]. In the present study, the elastic stiffness was determined from the slope of the linear region of the loading force–displacement curve. The reduction in elastic stiffness is defined by Equation (1)

**Figure 5:**
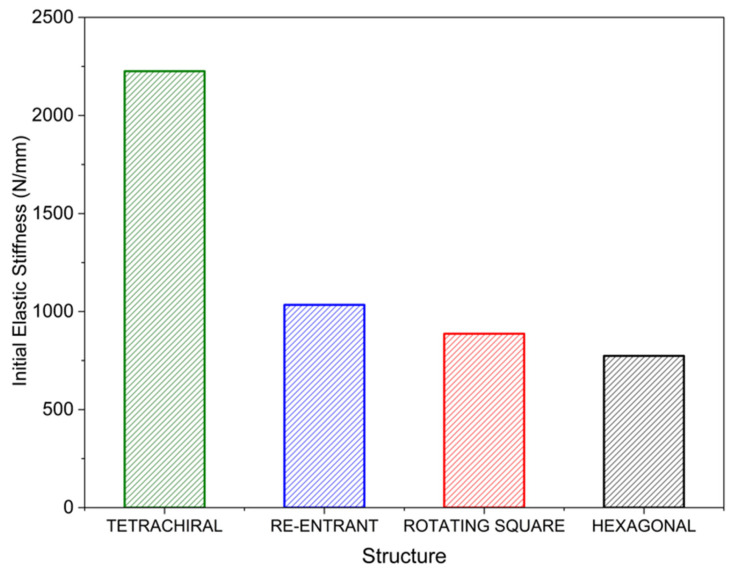
Initial stiffness of bone plates with different metamaterial structures.

Stiffness reduction (%) = (*E*_0_ − *E_cycle_*)/*E*_0_ × 100 (1)

where *E*_0_ and *E_cycle_* denote the initial elastic stiffness and the elastic stiffness at the current Cycle. The experiment was terminated when either a 60% reduction in the initial scaffold stiffness occurred or at the completion of 100,000 cycles, whichever came first. The initial elastic stiffness of each structure is given in Figure 5. Initial stiffness analysis showed that the tetrachiral structure possessed the highest stiffness under the applied load (~2225 N/mm), followed by re-entrant, rotating square, and hexagonal.

## 3. Results and Discussion

**Figure 6:**
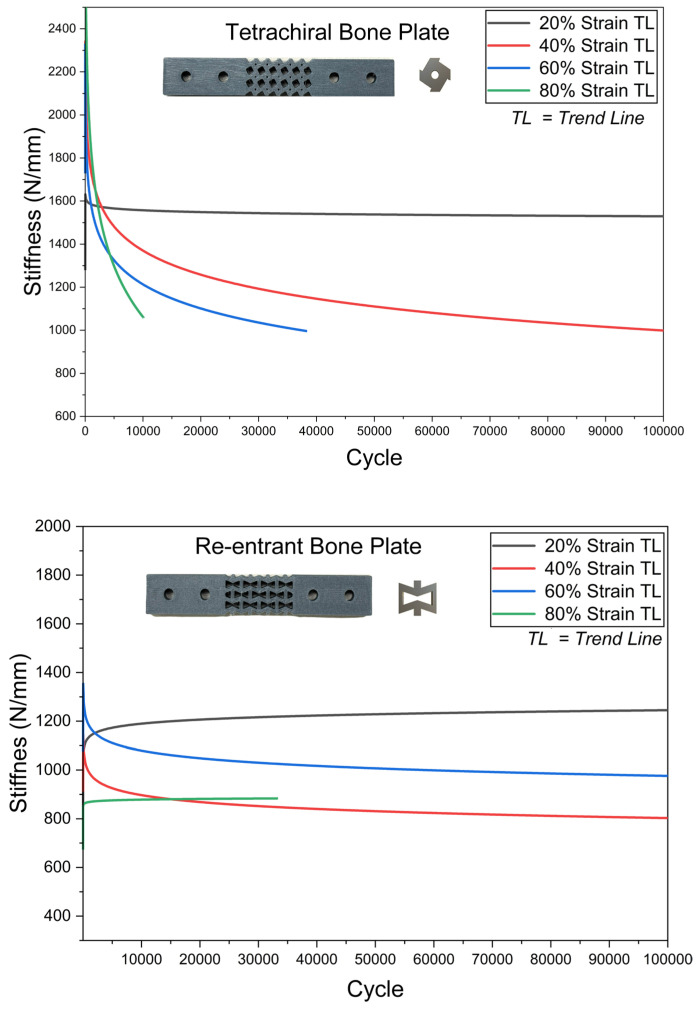

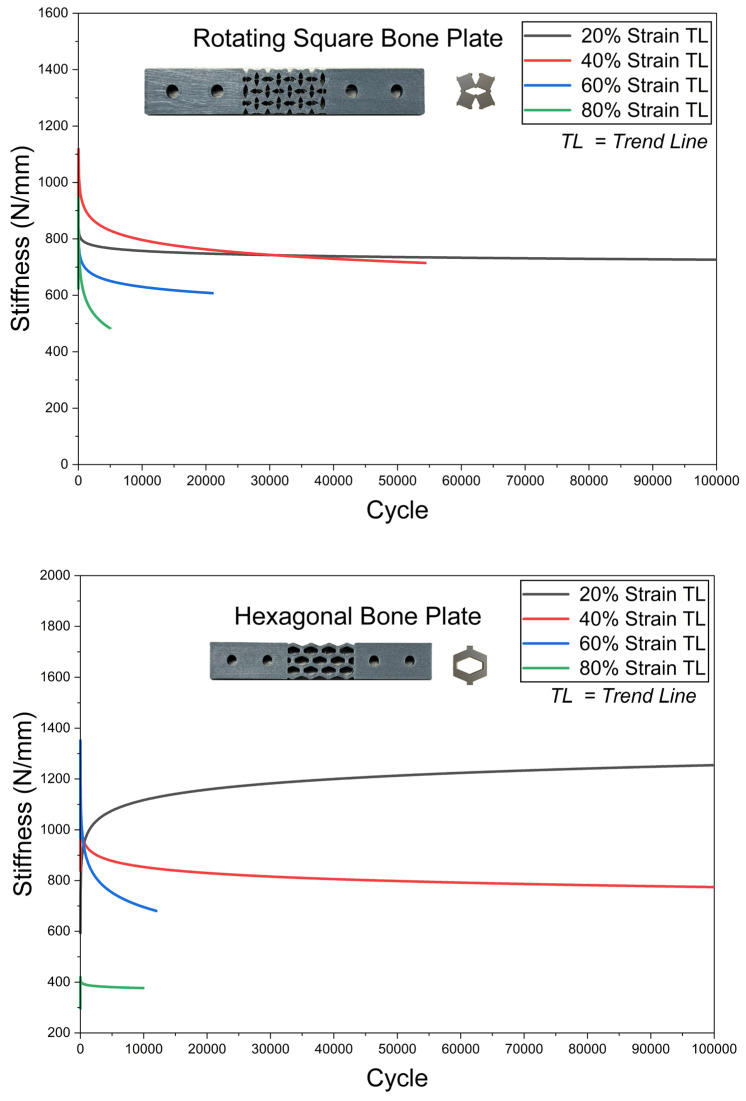
Fatigue behaviour of each metamaterial-structured bone plate.

**Figure 7:**
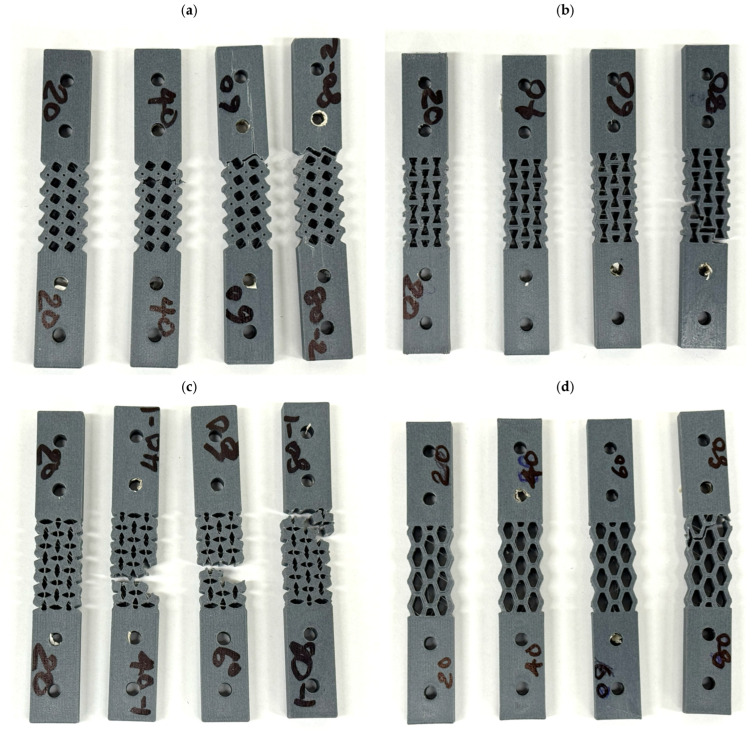
Bone plates after fatigue testing (**a**) tetrachiral, (**b**) re-entrant, (**c**) rotating square and (**d**) hexagonal. (20, 40, 60 and 80 represent the percentage strain levels at which the tests were conducted.)

The compressive fatigue behaviour of FDM printed CF-PLA bone plates for different auxetic structures, i.e., re-entrant, tetrachiral, hexagonal, and rotating square, have been examined for varying strain levels of 20%, 40%, 60%, and 80% over 100,000 loading cycles, as shown in Figure 6. All structures exhibited a characteristic fatigue response consisting of an initial rapid reduction in stiffness followed by a gradual stabilisation and progressive degradation with increasing number of cycles. Figure 7 shows the bone plates after fatigue testing.

As shown in Figure 6, at a strain level of 20%, all bone plate configurations successfully endured 100,000 loading cycles without failure. Notably, at this relatively low strain level, both the hexagonal and re-entrant structures exhibited an apparent increase in stiffness with continued cycling. Research has shown that early stages of cyclic loading may exhibit temporary stiffness increases due to micropore collapse and structural rigidity, followed by a decline as micro-damages accumulate [52]. Fatigue failure is commonly associated with the progressive accumulation of damage over repeated loading cycles. At low strain amplitudes, the damage incurred during each cycle is minimal and may not be sufficient to produce noticeable fatigue-induced changes in stiffness [60,77]. When it comes to 40%, excluding rotating square geometry, the remaining endured full cycles.

At higher strain levels (60% and 80%), all metamaterial bone plates exhibited pronounced stiffness degradation with increasing cycle count, although the extent of degradation and fatigue endurance varied significantly among the configurations. Higher loading amplitudes can induce greater deformation and plasticity in the polymer, leading to the development of additional defects and microcracks within the scaffolds. Over time, these imperfections may propagate and coalesce, ultimately leading to fatigue failure [78]. Gong et al. [79] demonstrated that increasing the strain amplitude from 0.7% to 3% resulted in a significant reduction in the fatigue life of both triangular and circular PLA scaffolds. At 60% strain, the re-entrant bone plate exhibited the best fatigue resistance, showing the slowest degradation and maintaining its stiffness throughout the full test duration. The tetrachiral structure experienced earlier fatigue softening, with a noticeable reduction in stiffness within the initial cycles. The hexagonal configuration degraded more rapidly, losing a significant portion of its stiffness within a relatively short number of cycles. In comparison, the rotating square structure showed the poorest performance, with early degradation and consistently low stiffness retention. At 80% strain, fatigue damage became significantly more severe for all configurations and failed before 100,000 cycles.

### Failure Mechanism

**Figure 8:**
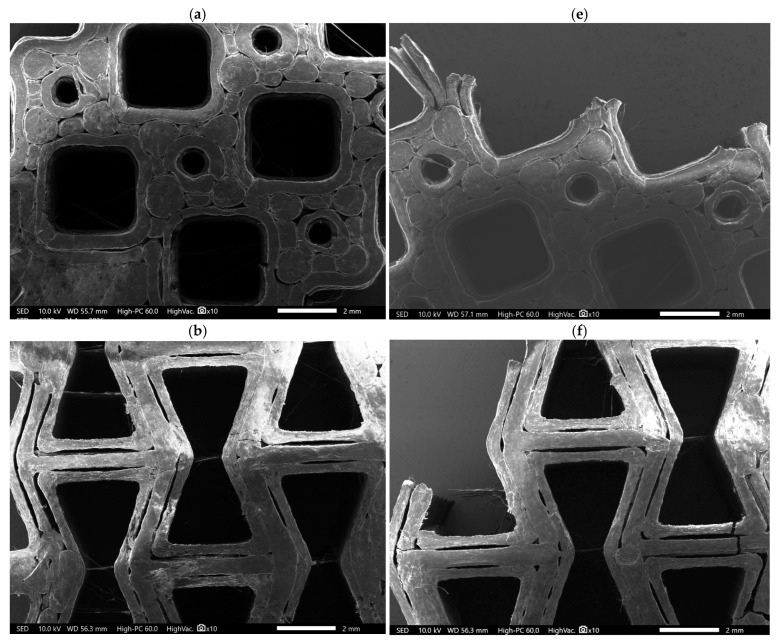

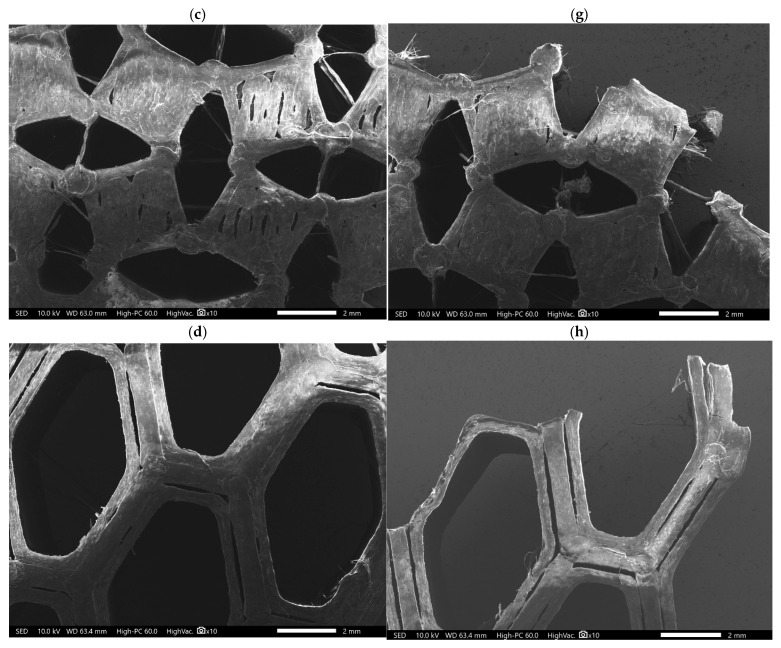
SEM images of bone plate metamaterial structures: (**a**) tetrachiral, (**b**) re-entrant, (**c**) rotating square, and (**d**) hexagonal; (**e**–**h**) corresponding fatigue-failed structures at a strain level of 80%.

Figure 8 shows the SEM images of each structure along with the corresponding failed segments following fatigue testing at an 80% strain level. All FDM-printed structures exhibited characteristic manufacturing defects, including poor interlayer adhesion and the presence of voids. The layer-by-layer addition of polymer in the FDM process imparts anisotropic material properties to the printed polymer, which affect the strength and fatigue performance of the printed components [36]. Under cyclic loading, repeated stress application accelerates damage accumulation. In particular, interlayer interfaces and inherent voids act as preferential sites for crack initiation and propagation, thereby contributing to the early onset of fatigue failure.

The re-entrant structure again demonstrated comparatively stable behaviour, sustaining stiffness for a longer duration than the other designs. Auxetic structures contract laterally under compression, effectively self-confining and densifying the structure. This behaviour delays the onset of local buckling and distributes strain more uniformly [80]. Re-entrant structures’ superior fatigue resistance is due to increased energy absorption with production angles and negative Poisson’s ratio [81]. Most research supports the superior fatigue performance of the re-entrant structure [81,82,83]. J. Michalski et al. [84] found that re-entrant cells exhibited significantly higher fatigue strength than regular hexagonal honeycomb. However, at the 80% strain level, the fatigue failure started at the sharp corners and eventually failed along the inter-layer defects that occurred during printing (Figure 8b,f). As shown in Figure 8b, interlayer defects observed in the re-entrant structure are primarily attributed to under-extrusion, which has caused delamination and failure of bone plates at 80% strain level. During compression fatigue, the sharp corners in re-entrant act as stress concentration zones and promote fatigue failure [55,85].

The compressive fatigue performance of tetrachiral can be attributed to its chiral cell topology, which provides a stable, ring-like structure to support loads. When compressed, the tetrachiral’s ligaments likely hinge in a manner that spreads the load uniformly through the lattice, delaying the onset of crushing or buckling [80,86]. At 80% strain level, despite its high initial stiffness, it exhibited rapid degradation within the early cycles. Failure in this structure is primarily governed by ligament bending and node rotation, with crack initiation occurring at the curved junctions, indicating significant stress concentration due to rotational deformation. This behaviour is evident from Figure 8e and is consistent with auxetic chiral systems reported to fail via node-dominated fatigue mechanisms [87].

The rotating square configuration, although inherently auxetic, exhibited failure at relatively low cycles, likely due to stress concentration effects or weak connectivity at the joints between the squares. Examination of the failed specimen (Figure 8c,g) confirms that the fracture initiated at the joints, corresponding to regions of maximum stress concentration. Rotating square metamaterials carry load primarily through rotation of the units, but the price is very high local stress at the interconnection regions. Previous studies have reported that certain planar auxetic geometries are susceptible to local buckling, which can limit their load-bearing capacity [88], which could also explain the early failure of the rotating square bone plate. Mizzi et al. [89] highlighted that rotating square lattices, despite their auxetic nature, tend to develop high localised stress intensities at nodal connections, resulting in low strain tolerance and early failure under applied loading.

In contrast, the non-auxetic hexagonal structure showed comparatively poor fatigue performance at higher strain amplitudes, surviving only about 12,000 and 900 cycles at 60% and 80% strain levels, respectively, although no failure was observed at lower strain amplitudes of 20% and 40%.

Similar to the re-entrant structure, poor interlayer adhesion and voids are clearly visible in the SEM image (Figure 8d). In the failed image (Figure 8h), the dominant feature is longitudinal splitting along a strut wall plus fracture centred around a multi-strut junction. This indicates that failure is primarily governed by layer separation rather than hinge-controlled failure. The non-auxetic hexagonal honeycomb bone plate expanded sideways under compression, which caused local buckling of the cell walls and reduced its load capacity. This observation is consistent with the findings of Francesconi et al. [65], who reported that auxetic structures exhibit superior fatigue life, exceeding that of conventional positive Poisson’s ratio (PPR) structures by more than 20% under identical loading conditions. The improved fatigue performance of auxetic designs is attributed to delayed crack initiation and a more uniform distribution of damage, resulting in a reduced stress intensity factor over a range of crack lengths [65].

**Figure 9:**
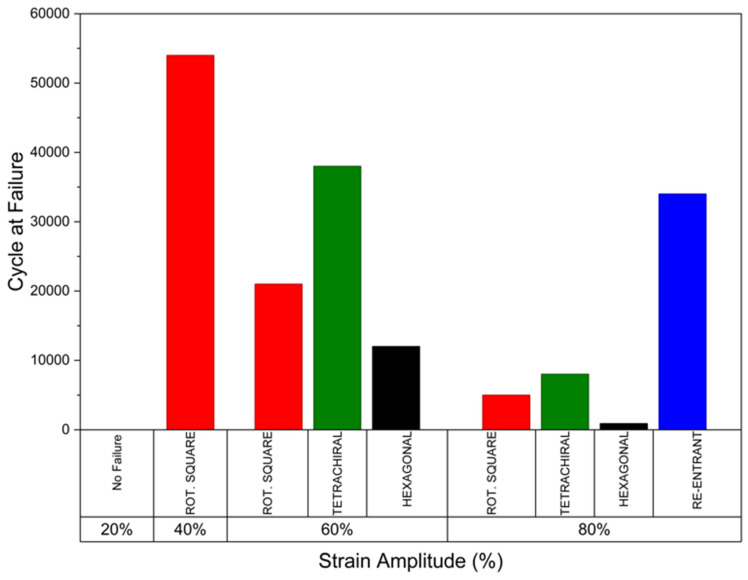
Fatigue failure cycle of each bone plate at different strain amplitudes.

Among the investigated designs (Figure 9), the re-entrant and tetrachiral structures exhibited the highest fatigue resistance. The re-entrant bone plate sustained 100,000 cycles up to 60% of its yield strain and only failed at the 80% strain level after approximately 38,000 cycles. The tetrachiral structure showed the second-best performance, withstanding approximately 38,000 and 8000 cycles at strain amplitudes of 60% and 80%, respectively. The rotating square structure exhibited moderate fatigue resistance, sustaining around 54,000, 21,000, and 5000 cycles at 40%, 60%, and 80% strain levels, respectively. Although the tetrachiral bone plate demonstrated favourable fatigue behaviour, its high compressive stiffness may limit its ability to elastically accommodate physiological bone strain in vivo, thereby potentially increasing the risk of stress shielding. Fu et al. [90] reported that increasing the ligament thickness relative to its length significantly increases the metamaterial’s stiffness. Tuning its stiffness would therefore be necessary to avoid over-constraining natural bone deformation. Compared to the other designs, the re-entrant bone plates may offer a more favourable performance due to their relatively lower stiffness combined with excellent fatigue resistance. This structure is capable of accommodating slight deformation under loading, allowing for improved stress distribution, while still maintaining sufficient mechanical strength to provide stable fracture fixation.

## 4. Conclusions

The study systematically evaluated the compressive fatigue behaviour of FDM-fabricated CF-PLA bone plates incorporating four metamaterial architectures, namely tetrachiral, re-entrant, rotating square, and hexagonal. The results showed that all designs survived 100,000 cycles at 20% strain, while at 40% strain, all configurations except the rotating square also completed the full loading regime. As the strain amplitude increased to 60% and 80%, stiffness degradation became substantially more pronounced, confirming that fatigue damage in these structures is strongly strain dependent. Among the investigated designs, the re-entrant structure demonstrated the best overall fatigue performance, surviving 100,000 cycles at 60% strain and failing only at approximately 38,000 cycles at 80% strain. The tetrachiral structure showed the second-best fatigue resistance, whereas the rotating square and non-auxetic hexagonal structures exhibited earlier failure, particularly at high strain amplitudes. These findings confirm that lattice architecture plays a critical role in governing stiffness retention, damage accumulation, and fatigue life in CF-PLA bone plates.

From a biomechanical perspective, the re-entrant design appears to be the most promising configuration for patient-specific bone fixation applications because it offers an effective balance between structural durability and reduced stiffness. Its relatively lower rigidity, combined with excellent fatigue resistance, may enable better stress distribution while still maintaining sufficient support for fracture stabilisation. Although the tetrachiral design exhibited favourable fatigue behaviour and the highest initial stiffness, its high compressive rigidity may increase the risk of stress shielding by limiting physiological strain transfer to the healing bone. In contrast, the poorer high-strain fatigue behaviour of the rotating square and hexagonal structures suggests that these geometries are less suitable for sustained load-bearing applications in their present form. Overall, this work provides a useful foundation for the design of fatigue-resistant, additively manufactured polymeric bone plates, and indicates that future optimisation should focus on tailoring lattice geometry to achieve both mechanical reliability and improved biomechanical compatibility.

## Figures and Tables

**Table 1:** Parameters of the metamaterial structures used in this study [4].

Type	Parameter	Value (mm)
Re-entrant	vertical length (h)	5.36
	inclined length (l)	4.18
	re-entrant angle (θ)	−20.00
	thickness (t)	1.56
	unit cell width (x)	7.85
	unit cell height (y)	7.85
Rotating squares	square side length (a)	3.01
	square rotation angle (θ)	45.00
	hinge offset distance (d)	0.80
	unit-cell width (x)	7.85
	unit-cell height (y)	7.85
Tetrachiral	ligament length (L)	6.64
	node radius (r)	2.10
	ligament thickness (t)	2.09
	ligament inclination angle (θ)	32.30
	cell width (x)	7.85
	cell height (y)	7.85
Hexagonal (non-auxetic)	vertical length (h)	2.50
	inclined length (l)	4.18
	inclined rib angle (θ)	20.00
	rib thickness (t)	2.03
	cell width (x)	7.85
	cell height (y)	7.85

**Table 2:** FDM process parameters for printing CF-PLA [76].

Parameters	CF-PLA
Nozzle Diameter	0.4 mm
Extrusion Multiplier	0.5
Initial Layer Height	0.2 mm
Layer Height	0.1 mm
Printing Speed	50 mm/s
First Layer Speed	50 mm/s
Extruder Temperature	230 °C
Bed Temperature	45 °C
Internal/External Fill Pattern	Rectilinear
Infill Percentage	100%

**Table 3:** Parameters of the metamaterial structures in the present study [4].

	Tetrachiral	Rotating Square	Hexagonal	Re-Entrant
0.2% Offset Yield Strength (MPa)	40	21	10.5	8.5
Offset Yield strain	0.017	0.014	0.013	0.013
Compressive Modulus (MPa)	2352.9	1500	807.7	653.8

## Data Availability

The original contributions presented in this study are included in the article. Further inquiries can be directed to the corresponding authors.
